# Patterns of Neurogenesis and Amplitude of Reelin Expression Are Essential for Making a Mammalian-Type Cortex

**DOI:** 10.1371/journal.pone.0001454

**Published:** 2008-01-16

**Authors:** Tadashi Nomura, Masanori Takahashi, Yoshinobu Hara, Noriko Osumi

**Affiliations:** 1 Division of Developmental Neuroscience, Center for Translational and Advanced Animal Research (CTTAR), Tohoku University School of Medicine, Sendai, Japan; 2 Core Research for Evolutional Science and Technology, Japan Science and Technology Agency, Kawaguchi, Japan; University of Washington, United States of America

## Abstract

The mammalian neocortex is characterized as a six-layered laminar structure, in which distinct types of pyramidal neurons are distributed coordinately during embryogenesis. In contrast, no other vertebrate class possesses a brain region that is strictly analogous to the neocortical structure. Although it is widely accepted that the pallium, a dorsal forebrain region, is specified in all vertebrate species, little is known of the differential mechanisms underlying laminated or non-laminated structures in the pallium. Here we show that differences in patterns of neuronal specification and migration provide the pallial architectonic diversity. We compared the neurogenesis in mammalian and avian pallium, focusing on subtype-specific gene expression, and found that the avian pallium generates distinct types of neurons in a spatially restricted manner. Furthermore, expression of *Reelin* gene is hardly detected in the developing avian pallium, and an experimental increase in Reelin-positive cells in the avian pallium modified radial fiber organization, which resulted in dramatic changes in the morphology of migrating neurons. Our results demonstrate that distinct mechanisms govern the patterns of neuronal specification in mammalian and avian pallial development, and that Reelin-dependent neuronal migration plays a critical role in mammalian type corticogenesis. These lines of evidence shed light on the developmental programs underlying the evolution of the mammalian specific laminated cortex.

## Introduction

The mammalian cortex is one of the most intricate brain structures in which distinct types of neurons are located in specific laminar positions enabling their elaboration into highly orchestrated neuronal circuits. (reviewed in [1]). Pyramidal neurons, ascending a thick apical dendrite towards the pial surface, are conspicuous constituents in the mammalian cortex (reviewed in [2]). During cortical development, all of pyramidal neurons are generated from two proliferative areas in the cortical primordium, the ventricular and subventricular zones. Neuronal progenitors in the ventricular zone, namely neuroepithelial cells or radial glial cells possess long processes that extend from the ventricular wall to the pial surface. These processes called radial glial fibers play essential roles for guiding neurons from the proliferative areas toward the pial surface (reviewed in [3], [4]). As corticogenesis proceeds, different types of pyramidal neurons are sequentially born to be fated to specific laminar positions. Newly generated neurons past earlier-born neurons to settle in more superficial layer; thereby the mammalian cortex is organized as an inside-out fashion. Spatio-temporaly cooperative regulation of neuronal specification and migration is thus essential for construction of the highly laminated mammalian cortex (reviewed in [5]–[7]).

The cortex is derived from the dorsal part of the embryonic telencephalon, called the pallium. The pallium is further subdivided into the medial, dorsal, lateral and ventral pallium, and these territories give rise to the hippocampus, neocortex, olfactory cortex and claustrum-amygdaloid complex, respectively [8], [9]. Recent studies have shown that remarkable conservation of the brain patterning across amniotes, a vertebrate group including reptiles, birds and mammals (reviewed in [10]). Comparative embryological analysis demonstrated conserved expression patterns of several transcription factors such as *Pax6*, *Emx1/2* and *Tbr1* in the dorsal part of the reptilian, avian and mammalian telencephalon, suggesting that those pallial regions are specified as the homologous territories in all amniotes [8], [11].

In contrast to marked homology of the pallial fields, neither six-layered laminated structures, nor mammalian-like pyramidal neurons are found in the dorsal part of the telencephalon of reptiles or birds, despite their common ancestry with mammals (reviewed in [1], [12]–[14]). Only three layers are present in the reptile pallium, but no laminar structures are evident in the avian pallium. Furthermore, the pallia of these species develop as outside-first to inside-last patterns [15]–[17]. Despite an enormous increase in knowledge on the molecular mechanisms underlying the mammalian cortical development, it remains unclear whether those mechanisms are also conserved in non-mammalian amniotes, and what kinds of developmental processes provide fundamental differences in morphology between the mammalian and non-mammalian pallia.

To address these questions, here we performed comparative experimental analysis of developmental processes in the avian pallium and the mammalian cortex. The avian pallium is also subdivided into several territories, in which distinct types of neurons are aggregated to form nuclear structures (reviewed in [18]). Comparison of distinct neuronal markers indicated that the expression of laminar specific genes was also detected in the avian pallium, although their expression patterns are largely different from those in the mammalian cortex. By cell tracing analysis, we identified that distinct neuronal subtypes are derived from distinct pallial ventricular regions in the avian telencephalon. Furthermore, we clarified that the number of Reelin-positive cells was smaller in the avian pallium compared with that in the mammalian cortex, and an experimental increase of Reelin-positive cells modified organization of the radial glial fibers, and consequently resulted in changes in the migratory modes of the avian pallial neurons from multi-polar to bipolar shapes. These data suggest that 1) distinct neuronal subtypes are generated in spatially regulated mechanisms in the avian pallium, and that 2) amplification of the number of Reelin-positive cells are essential for radial glial-dependent neuronal migration during mammalian cortical evolution.

## Results

### Expression patterns of mammalian layer-specific genes in the avian pallium

**Figure 1:**
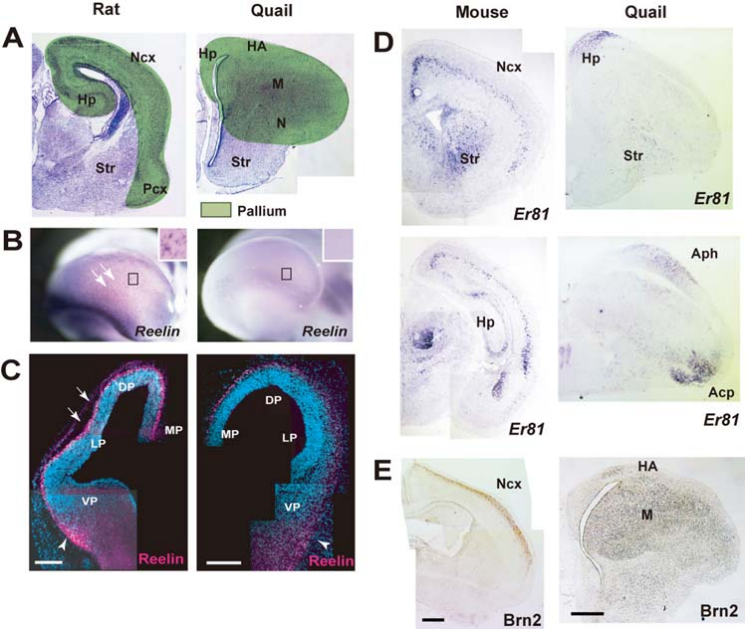
Distinct patterns of neuronal specification between the mammalian and avian telencephalon. (A) Coronal sections of P0 rat and quail telencephalon. The pallial region (green) can be compared as a homologous field in rodents and birds. Ncx, neocortex; Hp, hippocampus; Str, striatum; Pcx, piriform cortex. HA, hyperpallium apicale; M, mesopallium; N, nidopallium. (B and C) *In situ* hybridization with *Reelin* probe (B) and immunohistochemistry with anti-Reelin antibody (C) in the rat and quail telencephali. The number of Reelin-positive cells in the quail pallium (E4) is smaller compared with that in the rat pallium (E13.5). Arrows and arrowheads indicate Reelin-positive cells in the cortex and piriform cortex, respectively. MP, medial pallium; DP, dorsal pallium; LP, lateral pallium. (D and E) Expression patterns of *Er81* and Brn2 in the mouse (P4) and quail pallia (P0). *Er81* is expressed in the cortical layer V, hippocampus, amygdala and striatum, whereas Brn2 is mainly expressed in cortical layer II/III in the mouse telencephalon. In the quail telencephalon, *Er81* is expressed in the hippocampus, area parahippocampalis (Aph), arcopallium (Acp) and striatum. Brn2 expression is also detected in the quail hyperpallium apical (HA), mesopallim (M) and a part of nidopallium (N). Scale bars, 200 µm.

Recent molecular embryology has shown that similar to the mammalian cortex, a large part of the avian cerebrum is derived from the embryonic pallial regions [8], [11], indicating that this territory is comparable between the mammalian and avian telencephalon (Fig. 1A **)**. To compare specific neuronal subtypes between the mammalian and avian pallium, we first focused on several markers that are expressed in laminar specific subtypes of mammalian cortical neurons. The *Reelin* gene is robustly expressed in mammalian Cajal-Retzius cells, which are the most prominent cell population in the superficial layer of the mammalian cortex [19], [20]. In early-stage murine embryos, a large number of *Reelin*-positive cells were distributed on the entire surface of the pallium (Fig. 1B and 1C). However, in the developing quail embryos, we did not detect *Reelin* expression in the dorsal and lateral pallium, although a small number of *Reelin*-positive cells were localized on the surface of the medial pallium and the ventral part of the telencephalon (Fig. 1B, 1C and Figure S1), as reported previously in chick embryos [21]. We further examined distribution of other mammalian layer specific markers in the developing quail pallium. The gene *Er81* is specifically expressed in mammalian cortical layer V, which consists of early-born pyramidal neurons [22], [23] (Fig. 1D). In contrast, *Brn2* is expressed in cortical layer II/III and V, the former comprises later-born pyramidal neurons [22], [24] (Fig. 1E). Expression patterns of these genes in the quail pallium were extremely different from those in mammals: *Er81*-positive cells accumulated in the medial, caudodorsal and ventrolateral part of the quail pallium, corresponding to the hippocampus, area parahippocampalis, and arcopallium, respectively **(** Fig. 1D **)**. A large part of pallial regions including hyperpallium, mesopallium, and nidopallium is devoid of *Er81* expression. In contrast, Brn2-positive cells were distributed in the dorsolateral part of the pallium, corresponding to the mesopallium and nidopallium **(** Fig. 1E **)**. Thus, neurons express these markers are localized in discrete domains in the avian pallium, which are extremely different from those in the mammalian cortex.

### Origins of distinct neuronal subtypes in the developing avian pallium

**Figure 2:**
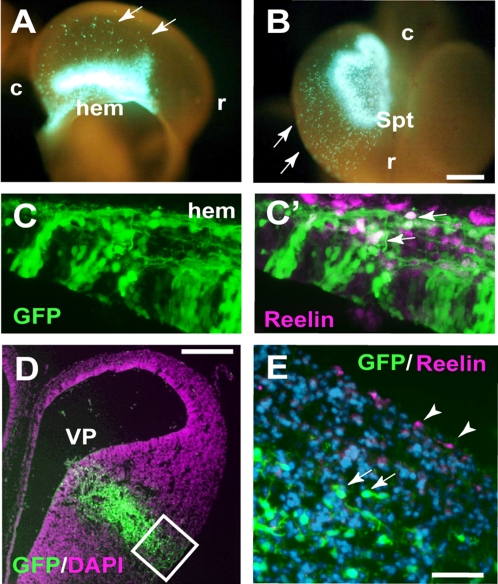
Origins of Reelin-positive cells in the developing quail telencephalon. (A and B) GFP-labeled cells in E4.5 quail telencephalon, in which GFP-expression vector was electroporated into the hem (A) or septum (Spt; B) at E3.5. Arrows indicate migrating GFP-positive cells. r: rostral, c: caudal. (C and C′) Expression of Reelin in the GFP-positive, hem-derived cells. (D) Coronal sections of the telencephalon in which GFP-plasmid was electroporated into the ventral pallium (VP). (E) The VP-derived GFP-positive cells (arrows) do not express Reelin. Arrowheads indicate Reelin-positive cells, which might be derived from the hem region. Scale bars, 200 µm (A, B, D), 50 µm (C, E).

To examine the developmental origins of *Reelin*, *Er81* and Brn2-positive neurons in the quail telencephalon, we performed cell-tracing analysis by focal electroporation of green fluorescent protein (GFP)-expression vectors. In the developing mammalian telencephalon, Reelin-positive Cajal-Retzius cells are originated from various telencephalic regions including the cortical hem [25], [26], septum [27], ventral pallium [27] and retrobulber regions [28]. Based on these lines of evidence, we focused on the quail hem, septum and ventral pallium, and examined whether these regions generate Reelin-positive cells. When we introduce the *GFP* gene into the E3 and E4 quail hem or septum, a large number of GFP-positive cells migrated on the surface of the quail telencephalon from electroporated regions (hem: n = 3, septum: n = 2, Fig. 2A and 2B). Immunohistochemical studies indicated that a subset of these GFP-labeled cells expressed Reelin (Fig. 2C). The migration patterns of these GFP/Reelin-positive cells were similar to those of mammalian Cajal-Retzius cells [25]–[27]; they migrate from medial to lateral regions of the telencephalon. However, when we labeled the quail ventral pallim by electroporation, generation of Reelin-positive cells was not detected from the labeled area (n = 3, Fig. 2D and 2E). To further confirm the results of *in vivo* tracing, we performed explant culture of distinct brain regions and examined Reelin expression. When we isolated E3 chicken cortical hem and cultured them for 48 hours, a large number of Reelin-positive cells differentiated in the explants (n = 8, Figure S2A, S2C and S2D). In contrast, we rarely detected Reelin-positive cells in explants taken from the ventral pallium (n = 8, Figure S2B, C and D).

**Figure 3:**
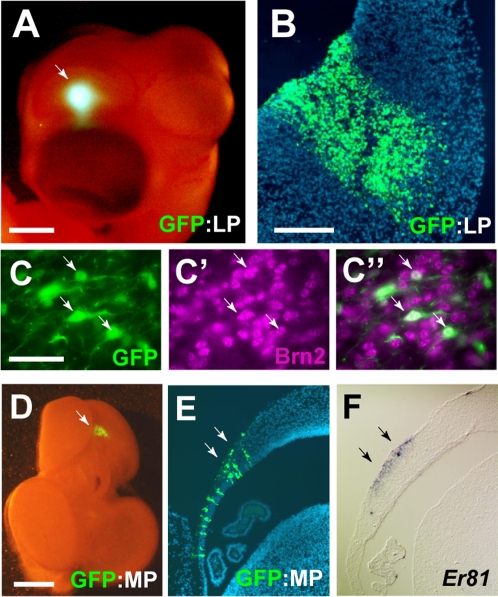
Origins of Brn2 and *Er81*-positive cells in the quail telencephalon. (A and B) Whole-mount (A) and coronal section (B) of E6 embryos in which GFP-expression vector were electroporated into the lateral pallium (LP). Arrow indicates the electroporated site. (C-C″) Immunohistochemistry indicates the LP-derived GFP-labeled cells express Brn2. (D–F) Whole-mount (D) and coronal sections (E and F) of the E7 telencephalon in which GFP was electroporated into the medial pallium (MP). The MP-derived GFP-positive cells (arrows) contribute to the *Er81*-positive region. Scale bars, 100 µm and 10 µm in whole mount and sections, respectively.

Next, we examined the origin of *Er81* and Brn-2 positive neurons in the developing quail pallium. Focal electroporation of *GFP* gene into the distinct pallial regions at E4 revealed that the nidopallial Brn2-positive neurons were derived from the lateral pallium (Fig. 3A–C), whereas the hippocampal and arcopallial *Er81*-positive neurons were originated from the medial and caudal parts of the ventral pallium, respectively **(** Fig. 3D–F, Figure S3 and data not shown**)**. In contrast to labeling the hem and septum, tangential neuronal migration was rarely observed in the case of labeling dorsal, lateral and ventral pallium at this stage.

**Figure 4:**
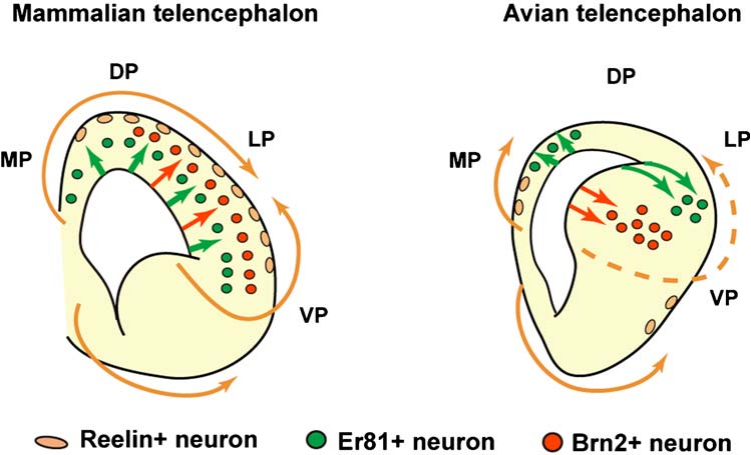
Schematic illustration of differences in neuronal specification and migration patterns between the mammalian and avian pallium. In the developing mammalian telencephalon, Reelin-positive neurons are derived from several origins including ventral pallium, and Er81 and Brn2-positive neurons are isotopically generated from entire pallial regions in a temporal manner. In contrast, in the developing avian telencephalon, Reelin-positive neurons are not derived from the ventral pallium, and Er81 and Brn2-positive neurons are generated from distinct pallial regions in a spatially regulated manner.

These data indicate that in the developing avian pallium, 1) Reelin-positive cells are few in number, and not derived from the ventral pallium, and that 2) *Er81* and Brn2-positive neurons are generated from distinct pallial regions in spatially restricted manner (Fig. 4). These features are markedly different from those in the mammalian cortex, in which a large number of Reelin-positive Cajal-Retzius cells is originated from several telencephalic regions, and *Er81* and Brn2-positive cortical neurons are isotopically generated from the entire cortical ventricular zone in a temporally regulated manner (Fig. 4). Thus, patterns of neuronal subtype specification in the avian pallium are largely different from those in the mammalian cortex.

### An experimental increase in Reelin-positive cells modified avian radial glial fibers

**Figure 5:**
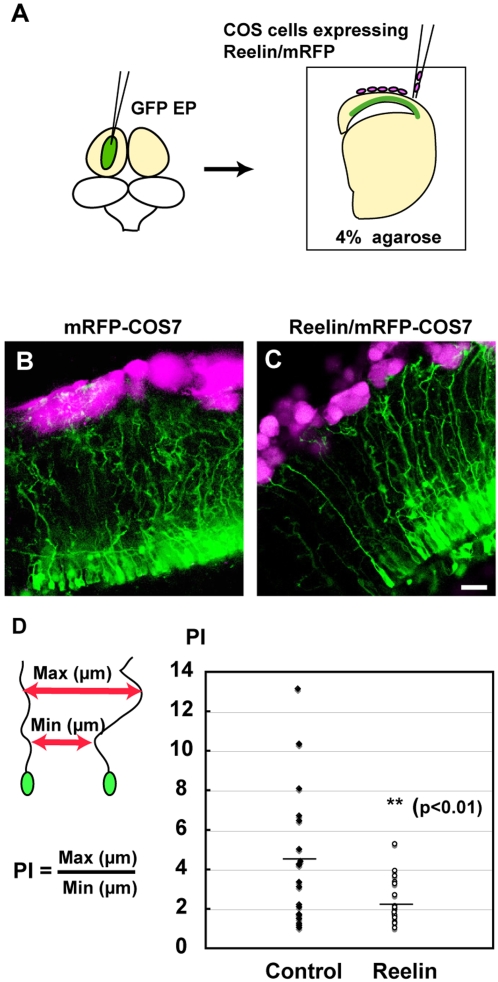
Reelin controls directed growth of radial fibers. (A) Schematic illustration of slice culture. After GFP plasmid electroporation, quail embryonic slices (E7) are co-cultured with Reelin and/or RFP-expressing COS7 cells (magenta). (B and C) Patterns of radial glial fiber extension in slices with control cells (B) and with Reelin-expressing cells (C). Straight projection of GFP-labeled radial fibers is evident with Reelin-expressing cells compared with control cells. (D) Quantification of radial fiber orientation. The parallel index (PI) of radial fibers was calculated by dividing the maximum distance between adjacent radial fibers by the minimum distance. Differences in PI between slices with control and Reelin-expressing cells were analyzed statistically (F-test). PI is reduced in Reelin-treated slices compared with that in control slices. Asterisks indicate statistically significance (p<0.01, F-test). Scale bar, 20 µm.

In the developing mammalian cortex, Reelin plays a crucial role in radial neuronal migration and the formation of laminar structures (reviewed in [29], [30]). Reelin is a large extra-cellular protein that is secreted from the Cajal-Retzius cells [31], [32]. The mice compromising generation of functional Reelin exhibit severe abnormalities in an inside-out pattern of corticogenesis; thereby six-layered laminar structure is disorganized (reviewed in [29], [30]). Previous results indicated that Reelin expression is also detected in the avian pallium, although their expression is less prominent compared to that in the mammalian cortex (Figure 1 and [21]). Hence, it is possible that some, if not all, of the architectural differences between the mammalian and avian pallium, might be due to differences in the amplitude of Reelin signaling in these taxa. In order to test this hypothesis, we examined the effect of experimental amplification of Reelin signaling on avian pallial development, by co-culture of the quail telencephalon (E7) with COS7 cells transfected with a *Reelin*-expression vector (Fig. 5A). To trace immature neuronal progenitors and migrating neurons, a *GFP* expression vector was electroporated into the slice. Although we did not detect significant changes in neuronal migration patterns in this culture, we identified significant alterations in the attachment of radial glial cells (radial fibers) to Reelin-expressing cells. In the slices with control COS7 cells, GFP-labeled radial fibers did not extend straight, but exhibited curled morphology in the neuronal layer (Fig. 5B). We identified a similar projection pattern of radial fibers samples *in vivo* **(** Figure S4 **)**, indicating that this effect is not artifactual to the culture conditions. Labeling radial fibers with specific markers, DiI or GFP in fixed samples indicated a meandering extension of radial fibers in the quail pallium (Figure S4B–D). This is extremely different from the organization of mammalian radial glial fibers, which project straightly from the ventricular zone toward the pial surface (Figure S4A). However, when the quail slices were co-cultured with Reelin-expressing COS cells, GFP-labeled radial fibers became to extend long processes in highly parallel orientation towards the pial surface (Fig. 5C). To quantify the alteration in radial fiber organization, we determined the “parallel index” of fibers by calculating the ratio of the maximum to minimum distances between two fibers (Fig. 5D). In control cultures, the parallel index ranged from 1.2 to 13.1 (n = 22, 3 slices), indicating that radial processes were oriented randomly. In contrast, the parallel index was significantly lower in cultures containing Reelin-expressing cells (ranged from 1.09 to 5.34, n = 20, 3 slices), indicating that fibers extended with less directional variance than in controls (Fig. 5D). These data indicate that 1) projection patterns of radial glial fibers in the quail pallium is largely different from those in the mammalian cortex, and that 2) exogenous Reelin modified extension patterns of the quail radial fibers as those seen in the mammalian cortex.

### An increase of Reelin-positive cells in the avian pallium by *Dbx1* overexpression

**Figure 6:**
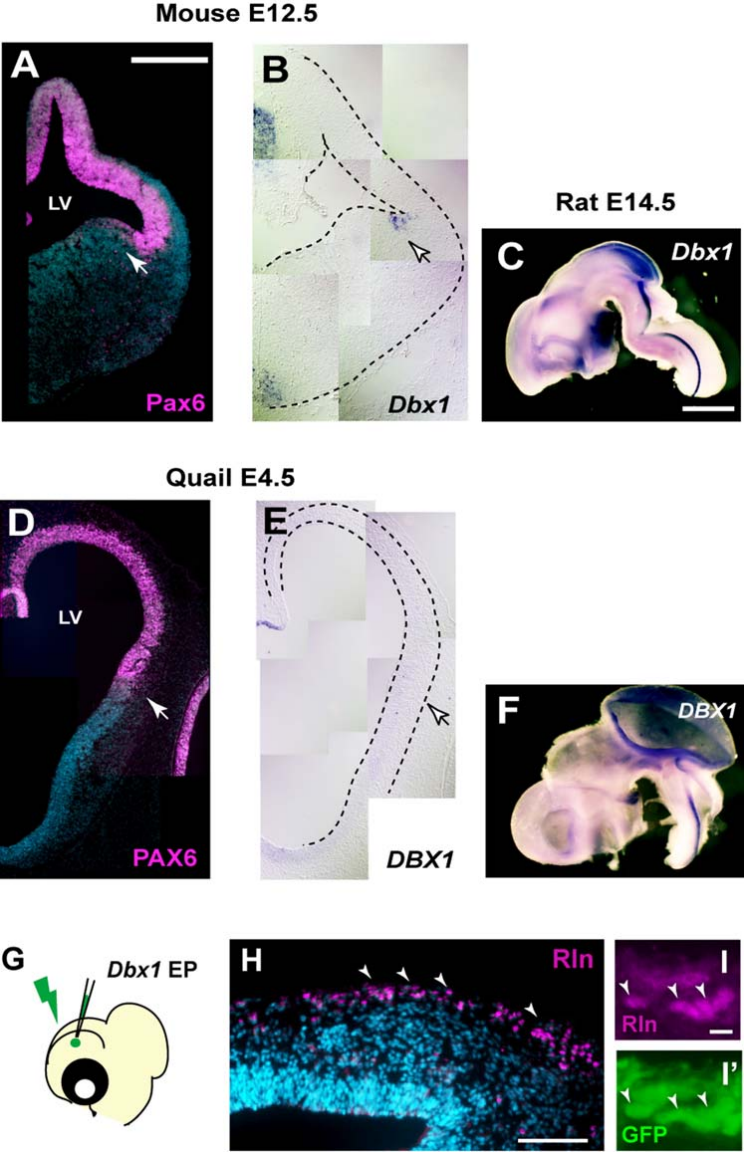
Induction of Reelin-positive cells in the quail pallium by *Dbx1.* (A–F) Expression patterns of Pax6/PAX6 and *Dbx1/DBX1* in E12.5 mouse (A and B), E14.5 rat (C) and E4.5 quail (D–F) embryos. Immunostaining with anti-Pax6 (A) and PAX6 (D) antibodies reveal pallial regions in these species (arrows in A, B, D and E). *In situ* hybridization with rat *Dbx1* (B and C) and quail *DBX1* (E and F) probes demonstrate expression of *Dbx1* in the mouse ventral pallium (B) but not in the quail ventral pallium (E). (G–I) Induction of Reelin-positive cells in the quail pallium by overexpression of *Dbx1*. *Dbx1* expression vector is electroporated at E4 embryos (G). After 3 days of electroporation, a large number of Reelin-positive cells are generated in the quail pallium (H). All of Reelin-expressing cells are positive for co-electroporated GFP (I and I′). Scale bar: 200 µm.

Since massive neurogenesis and neuronal migration in the avian pallium take place at early embryonic stages [16], [33], it is possible that the onset of the culture was initiated too late to examine the effect of Reelin on neuronal migration. In order to overcome these problems, we tried to increase Reelin-positive cells from early stages by *in ovo* electroporation. Unfortunately, we failed to introduce Reelin-expression vector into the quail pallium, probably owing to the large size of the plasmid (16 kb), and so adopted an alternative strategy to increase Reelin-positive cells. A previous study showed that *Dbx1* gene is expressed in the mammalian ventral pallium, which generates a subset of Reelin-positive Cajal-Retzius cells ([27] and Fig. 6B). Furthermore, it has been shown that the expression of *Dbx1* is not detected in the developing chicken ventral pallium [27]. We also confirmed that the quail ortholog of *Dbx1* (*DBX1*) is not expressed in the ventral pallium, although strong expression was detected in other brain regions (Fig. 6D–F). Considering together with the evidence that the avian pallium did not generate Reelin-positive cells (Fig. 2 and Figue S2), these data suggest that *Dbx1* plays a key role in the generation of Reelin-positive cells across vertebrate species. To examine this hypothesis, we overexpressed *Dbx1* by introducing an expression vector into the E4 quail pallium (Fig. 6G). After 3 days of electroporation, we found that a large number of Reelin-positive cells were induced in the quail pallium **(** Fig. 6H and 6I). The newly generated Reelin-positive cells accumulated preferentially on the pial surface of the quail pallium, as Cajal-Retzius cells in the mammalian cortex (Fig. 6H). Induction of Reelin-positive cells by *Dbx1* overexpression was detected various pallial regions, suggesting that a broad area of the quail pallium has a competence to generate Reelin-positive cells. These data indicate that *Dbx1* has a potential to induce Reelin-positive cells, and that overexpression of this gene provides a recapitulation of the distribution of the Cajal-Retzius cells in the developing avian pallium.

### Morphological changes in the migrating neurons in the avian pallium by *Dbx1* overexpression

**Figure 7:**
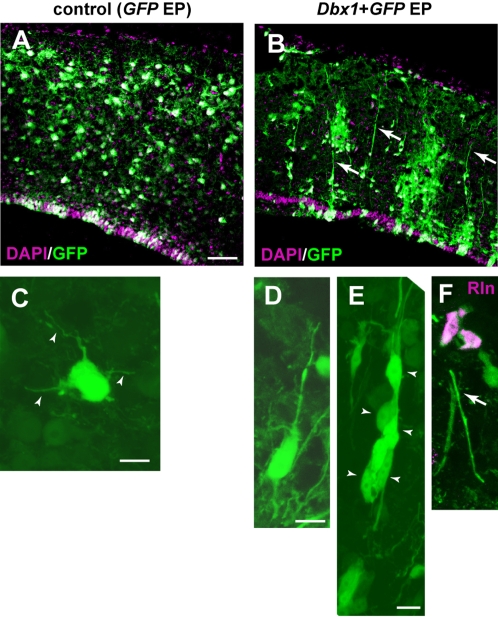
An increase of Reelin-positive cells changes morphology of migrating neurons. (A and B) Changes in radial fiber projection patterns in E10 telencephali by *Dbx1* misexpression. Compared with control (A), straight projection of radial fibers is evident in the *Dbx1*-misexpressed pallium (arrows in B). (C–E) Morphological changes in migrating neurons by *Dbx1*-misexpression. In control, GFP-labeled migrating neurons extend many short processes (arrowheads in C). In contrast, In *Dbx1*-misexpressed pallium, migrating neurons display a bipolar shape (D), and attached to the radial fibers (arrowheads in E). Each radial fiber extends to the Reelin-positive cells localized at the pial surface (an arrow in F). Scale bars, 50 µm (B), 20 µm (D), 10 µm (F, G).

To address whether an increase in Reelin-positive cells alter neuronal migration in the developing quail pallium, we examined stage-dependent neuronal distribution in *Dbx1* mis-expressed pallium. After electroporation of *Dbx1* expression vector, we injected small amount of BrdU-containing solution into the lateral ventricle at different embryonic stages (E4 or E7), and collected the embryo at E10 (Figure S5A, S5D). As previously reported in chick embryos [16], [17], the quail pallium develops roughly in an outside-in fashion; the cells born at early stages are distributed in both superficial and deep areas of the pallium, whereas those born at later stages are located specifically on deep side of the pallium (Figure S5B and S5E). Although the number of Reelin-positive cells was increased in the *Dbx1*-misexpressed embryos, distribution of BrdU-positive cells was not significantly changed compared with control embryos (Figure S5C, S5F S5H and S5I). In contrast, *Dbx1*-overexpression increased the number of GFP-positive cells in the ventricular zone (Figure S5J). Furthermore, we identified remarkable alterations in radial fiber organizations and neuronal migration patterns in the *Dbx1*-overexpressed embryos (Fig. 7). In control embryos, the radial fibers extended in a meandering fashion, and almost all of migrating neurons exhibited a multi-polar morphology, characterized by a round soma and extending short processes during migration (Fig. 7A and 7C). In contrast, the straight elongation of radial fibers was clearly evident in the *Dbx1* misexpressed pallium (Fig. 7B). These radial fibers were also immunoreactive for Transitin, indicating that they are processes of immature/progenitor cells (Figure S6). Furthermore, significant numbers of GFP-positive migrating cells exhibited a bipolar shape, and attached to the radial fibers **(** Fig. 7D and 7E). Immunohistochemical study revealed that these bipolar shaped cells expressed a neuronal marker Tuj1 (Figure S7). We also confirmed that the radial fibers consistently projected to the Reelin-positive cells located in the pial surface in the *Dbx1*-misexpressed pallium (Fig. 7F). These results indicate that an increase of Reelin-positive cells by *Dbx1* overexpression altered the morphology of migrating neurons via modification of the radial fibers.

## Discussion

In the present study we revealed that transcription factors expressed in the mammalian layer-specific neurons are also expressed in discrete regions in the avian telencephalon, and that they are specified in a spatially regulated manner. *Er81*, a marker of mammalian layer V neurons, is expressed in the hippocampus, area parahippocampalis and arcopallial regions, whereas Brn-2, a marker of layer II/III and V neurons, is detected in the mesopallial and nidopallial regions of the avian telencephalon. Curiously, it was reported that the avian arcopallium contains extra-pallial projection neurons, similar to those observed in mammalian cortical layer V [34]. Moreover, the avian nidopallium has intra-pallial projection neurons, as in the case of the mammalian layer II/III [35]. Thus, there are marked similarities between the avian and mammalian telencephalon in both their gene expression patterns and neuronal connectivity, as previously suggested (reviewed in [18]). However, there is still an argument which of mammalian pallial region is homologous to the avian arcopallium. Based on comparative neuroanatomical and experimental embryological studies, it has been suggested that the avian arcopallium is the homologue of a part of mammalian amygdala [8], [36], [37]. We do not exclude this possibility since *Er81* is also expressed in the basolateral nucleus of the amygdala (Fig. 1). In addition, not all gene expression patterns exhibit one-to-one correlations between the mammalian cortical layers and avian pallial divisions [38]. Further analysis on detailed comparative gene expression studies might provide more solid conclusions on homology of the mammalian and avian pallial structures.

It has previously been proposed that the avian pallium is organized as a developmental “compartment”, in which distinct types of neurons are distributed perpendicular to the pallial ventricular zone [reviewed in 39]. This is originally based on the protomap hypothesis on the mammalian cortex, in which proliferative units of progenitors in the ventricular zone is translated into the ontogenic cortical columns [40]. Our results on gene expression and cell tracing analysis are strongly consistent with these hypotheses, and further indicate that a genetic program that resides in the avian pallial neuroepithelial cells establishes this neuronal compartment. Previous studies have shown that a variety set of transcription factors play pivotal roles in the establishment of brain compartment (reviewed in [41], [42]). Furthermore, expanding sets of genes with neuronal specification have recently been identified in the developing mammalian cortex (reviewed in [7]). These lines of evidence suggest that evolutionary conserved genetic program control the spatial or temporal regulation of neurogenesis in distinct styles of pallial development.

**Figure 8:**
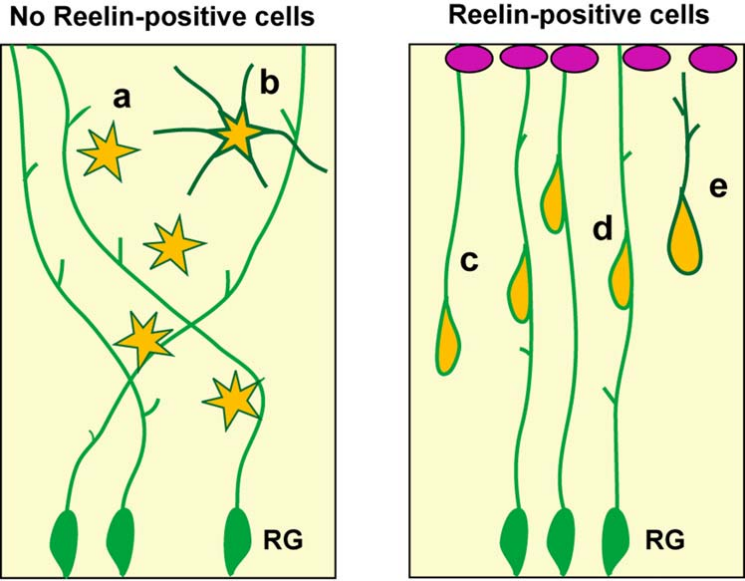
A role of Reelin-positive cells in the developing vertebrate pallium. (Left) Without Reelin-positive cells, radial glial cells (RG) extend fibers in multiple orientations. Neurons migrate independently, and exhibit a multi-polar shape (a, b). (Right) In the presence of Reelin-expressing cells at the pial surface (magenta), RG fibers are directed toward the source of Reelin. Migrating neurons shorten their own fibers for translocation (c) or attach to RG fibers for locomotion (d). Neurons exhibit a polarized shape during and after migration (e).

In the developing mammalian cortex, parallel elongated radial fibers play essential roles in radial neuronal migration, by serving as a migratory scaffold or an anchor for translocation, thereby giving rise to the columnar distribution of pyramidal neurons (reviewed in [3], [4], [43]). Several studies demonstrated that Reelin signaling regulates the extension and orientation of radial fibers [44], [45]. In the hippocampus of Reelin*-*signal deficient mice, radial fibers in the dentate granule cell layer randomly project, and a laminar structure is severely disrupted [45], [46]. However, exogenous Reelin refined radial fiber alignment as seen in normal mice, thereby laminar organization was restored [45]. Thus, straight extension of the radial fiber is prerequisite for laminar formation during mammalian brain development. It is not yet possible to state conclusively whether the unique feature of avian radial fibers is due to the absence of Reelin signaling or other unknown mechanisms. However, the present study provides significant evidence of the role of radial fibers in the pallial development across vertebrate species: contribution to the morphological conversion of migrating neurons. During mammalian cortical development, multi-polar to bipolar conversion is an essential step for migrating neurons to reach out the cortical plate [47], and to establish a highly polarized “pyramidal” shape [48]. In contrast, the avian pallial neurons always display multi-polar morphology, retaining symmetrical dendritic trees, during and after migration. Concomitantly, histological evidence indicates that the avian pallium is devoid of pyramidal neurons (reviewed in [13]. We propose that Reelin-dependent directed growth of radial fibers substantially contribute to the mammalian specific “pyramidal” shape of neurons (Fig. 8), in addition to direct roles of Reelin on the migrating neurons themselves [49], [50], and/or another cell intrinsic/extrinsic mechanisms for establishment of the neuronal polarity (reviewed in [51]). Although we could not eliminate a possibility that the morphological change of radial glial cells is due to secondary effects by Reelin or *Dbx1* overexpression, future experiments such as functional blocking of Dab1 protein, which is an intracellular mediator of Reelin signaling [52], will clarify direct/indirect influences of Reelin on radial glial fibers.

Although overexpression of Reelin or *Dbx1* drastically altered the morphology of the radial scaffold, we were not able to detect significant changes in neuronal positioning during avian pallial development. Since morphological changes of radial fibers occurred only in limited number of progenitor cells, it might be insufficient to induce overt rearrangement of neuronal distribution. Alternatively, it is possible that the radial glial scaffold is a necessary but not sufficient component to establish inside-out patterns of neuronal migration. Interestingly, the avian optic tectum develops in not-inside-out fashion, although radial fibers straightly extend to the pia [53]–[55]. These evidences suggest that additional mechanisms are required for inside-out corticogenesis, which might be specifically acquired during mammalian cortical evolution.

Comparative analysis of *Reelin* expression in distinct amniotes implied that massive amplification of *Reelin* expression occurred in the mammalian cortical marginal zone (reviewed in [56]). Thus, the regulation of *Reelin* expression might have been altered during mammalian cortical evolution. We have shown a causal relationship between *Dbx1* expression and the generation of Reelin-positive cells in non-mammalian species. Recent studies have shown that Reelin-positive Cajal-Retzius cells are heterogeneous and originated from distinct telencephalic regions [25]–[28]. Comparative gene expression analysis in Cajal-Retzuis cells between reptiles and mammals suggested that the cortical hem is rudimentary in reptiles, which might contribute to small number of Reelin-positive cells in this species [57]. Our data also suggest that acquiring of *Dbx1* expression in the ventral pallium plays a key role in the increase of Reelin-expressing cells in the mammalian cortical evolution, in addition to other regulatory systems for induction of *Reelin* gene in various telencephalic regions [58], [59]. Furthermore, since Reelin is weakly expressed in the avian pallium, especially at later embryonic stages (Figure S1 and ref. 21), blocking endogenous Reelin function will also provide significant evidence on the roles of Reelin signaling across vertebrate species.

Taken altogether, the present study indicates two fundamental developmental mechanisms underlying distinct styles of brain architectures: 1) Patterns of neuronal specification are essential for building isotopic laminated sheets or parcellated nuclear structures, and 2) Reelin-dependent radial fiber organization is required for the bipolar migratory shape of neuron. These results support the idea that the evolutionary novelties in the cortex might be provided by relatively small genetic differences effecting the timing or level of gene expression during early embryogenesis [60]. Coupled with our growing knowledge of the cortical development, experimental approaches in distinct vertebrate species, as shown here, could further shed new light into the cellular and molecular programs that underlie the evolution of our mammalian-type cerebral cortex.

## Materials and Methods

### Animals

Fertilized Japanese quail and chicken eggs were obtained from a local farm (Sendai Poultry Farming, Ova production in Sweden). The eggs were incubated under high humidity at 38°C until surgical manipulation. Pregnant Sprague-Dawley (SD) rats and CD-1 mice were purchased from Japan Charles River (Tokyo, Japan). Experimental design for embryonic manipulation has been provided in Supplementary Information. The committee for animal experiments of Tohoku University Graduate School of Medicine approved all experimental procedures.

### Explant culture in Matrigel

The cortical hem and ventral pallium were dissected out from E3 chicken embryos and cultured in Matrigel (BD Bioscience) containing with Dulbecco's modified Eagle's medium (DMEM) containing 10% fetal calf serum (FCS). The explants were cultured at 37°C under 5% CO_2_ for 48 hours.

### Slice culture with Reelin-expressing COS7 cells

pCDNA3-*reelin* (pCrl, a kind gift from Dr. T. Curran) and/or pCAGGS-*mRFP* (a kind gift from Dr. M. Uchikawa) were transfected into COS7 cells by using Lipofectoamine 2000 (GIBCO, BRL) 1 day before performing slice culture. Reelin expression in COS cells was confirmed by immunohistochemistry with anti-Reelin antibody CR-50 (not shown). To prepare brain slices, the dissected quail telencephalon was embedded in 4% low-melting agarose (Cambrex Bioscience), and 400 µm sections were prepared using a vibratome (Microslicer, Dosaka). Sections were transferred on collagen-coated membrane (Coaster 3492), and cultures were supplied with DMEM containing 10% fetal calf serum (FCS). After 6 hours in preculture, pCrl and/or pCAGGS-*mRFP*-transfected COS7 were collected and plated with a glass needle on the side of pial surface of the slices. Slices with COS7 cells were cultured at 37°C under 5% CO_2_ for 2 days.

### Isolation of quail *DBX1* and mouse *Er81*

A partial quail *DBX1* clone was amplified by PCR from quail genomic DNA by using specific primers for first exon of chicken *Dbx1* based on chicken genomic information (NCBI Chicken Genome Resources). Sequences of primers are 5′-ATG ATG TTC CCC AGC CTC AT-3′ and 5′-TTG ACC CCG AAC TTG AGG AAA-3′. The isolated clone was 96% identical to the chicken *DBX1* first exon based on nucleotide sequence (GenBank accession no. XM416044). The cDNA fragment of mouse *Er81* (GenBank accession no. L10426) was also amplified by PCR. Sequences of primers are 5′-TCT AGA GAT GGA TTT TAT GAC CAG CAA G-3′ and 5′-GTG CCT TGT TTG ACG GGT TAC-3′.

### *In situ* hybridization

Digoxygenin (DIG)-labeled cRNA probes were prepared by DIG RNA labeling kit (Roche Molecular Systems) from mouse *Reelin* (from Dr. M. Ogawa), chick *Reelin* (from Dr. H. Nakamura), chick *Er81* (from Dr. H. Nakamura), mouse *Er81*, quail *DBX1*, rat *Dbx1*cDNAs [61] that were subcloned into pBluescript II (Stratagene). The hybridization procedures were as described previously [61].

### Immunohistochemistry

Embryos were fixed with phosphate buffered saline (PBS) containing with 4% paraformaldehyde and sectioned with cryostat (CM 3050, Leica Instruments). Sections were washed with Tris-buffered saline plus 0.01% Tween 20 (TBST) and were incubated overnight with CR-50 anti-Reelin [32], anti-pan vertebrate Reelin (Chemicon International, Inc.), anti-mouse Pax6 [62], anti-chicken PAX6 (Developmental Studies Hybridoma Bank), anti-BrdU (BD Bioscience), anti-acetylated ß-III tublin (Tuj1, Covance), anti-GFP (Abcam) or 7B3 anti-transitin [63] antibodies. Cy3 or Alexa Fluor 488 conjugated secondary antibodies (Jackson Immunoresearch Laboratories) were applied to the sections. Co-staining with anti-Reelin antibody and Tuj1 was performed with Zenon Mouse IgG Labeling Kits (Z25102, Molecular Probes). After rigorous washing with TBST, the sections were examined by fluorescent microscopy (Axioplan2, Zeiss) and images were captured using a cooled CCD system. The arrangement of radial fibers was examined with laser confocal system (LSM5-PASCAL, Zeiss).

### Embryonic manipulation

Experimental procedures for surgical manipulation and electroporation were described previously [64]–[66]. For electroporation, after injection of DNA solution containing plasmid vector pCAX-*GFP* [61] and/or pCAX-Flag-r*Dbx1*1into the quail lateral ventricle, square pulses (35 V, 50 ms, 3 pulses) were applied to the pallium by a forceps-type electrode. For BrdU pulse labeling of neurons, 0.2–0.5 µl of solution containing 40 µM BrdU was injected into the lateral ventricle. Manipulated embryos were incubated at 38°C in high humidity.

## Supporting Information

Figure S1 Expression patterns of Reelin in E10 quail pallium. (A) Coronal sections of the quail telencephalon illustrating Reelin expression (magenta). (B) Immunostaining with anti-Reelin antibody in E10 quail telencephalon. Arrows indicate Reelin-positive cells distributed at the pial surface. In later stages, mitral cells in the olfactory bulb (OB), and some neurons in the hyperpallium apicale (HA) and hippocampus (Hp) become to express Reelin, as previously reported in chick embryos [21]. APH: area parahippocampalis. Scale bar: 50 µm.
(1.28 MB TIF)

Figure S2 Reelin expression in explant culture. (A, B) Matrigel culture of the E3 chicken cortical hem (A) and ventral pallium (B). After 48 hours of culture, cells migrate out of the hem (inset in A), but not out of the ventral pallium (B). (C) Immunostaining of explants with anti-Reelin and anti-β III tubulin antibodies. (D) The number of Reelin-positive cells in explants. Compared with hem explants, Reelin-positive cells are rarely appeared in the ventral pallium explants. Asterisks indicate statistical significance (p<0.01, *t*-test).
(2.03 MB TIF)

Figure S3 Expression of *Er81* in the medial pallium-derived cells. (A–E) Expression of GFP (A, C) and *Er81* (B, D) in an E10 embryo in which GFP-plasmid is electroporated into the medial pallium. In situ hybridization with *Er81* probe and immunohistochemistry with anti-GFP antibody are performed on same sections.
(2.40 MB TIF)

Figure S4 Curved projections of radial glial fibers in the developing quail pallium. (A) Immunostaining with anti-Nestin antibody indicating straight extension of the mouse radial fibers. (B) Immunostaining with anti-Transitin antibody, recognizing an intermediate filament, reveals a mesh-form organization of the quail radial fibers. DiI labeling shows curved extension of each radial fiber in the developing quail pallium. VZ, ventricular zone. (C and D) An image of radial fibers in a flat-mounted quail brain. Confocal microscopic analysis from the pial surface shows multi-directional extension of radial fibers in the developing quail pallium (arrowheads in D). Scale bars, 100 µm.
(1.36 MB TIF)

Figure S5 No alterations in birth date-dependent neuronal distribution by *Dbx1* overexpression. (A, D) Schematic illustration of schedules of BrdU pulse labeling in electroporated embryos. (B, C, E, F) Distribution of BrdU-positive cells labeled at E4 (B and C) or E7 (E and F) in control (B and C) and *Dbx1* overexpressed pallia (E and F). In both cases, the cells incorporated BrdU at E4 are distributed in superficial and deep pallial areas (B and C), whereas the cells labeled at E7 are localized at deep pallial areas (E and F). (H–J) Distribution of BrdU- (H, I) and GFP-positive (J) cells in the control and *Dbx1* -overexpressed pallia. No significant changes in the distribution of BrdU-positive cells between the control and *Dbx1* -overexpressed pallia (H and I). In contrast, *Dbx1* oveexpression increased the number of GFP-positive cells in the ventricular zone (J, BIN1). Asterisk indicates statistical significance (p<0.05, *t*-test). Scale bar, 100 µm.
(1.53 MB TIF)

Figure S6 Expression of progenitors and neuronal markers in *Dbx1* overexpressed embryos. (A–H) Immunostaining with anti-Transitin (B, D) or anti-β III tubulin (F, H) antibodies of control (A, B, E, F) and *Dbx1*-overexpressed pallia (C, D, G, H). Elongating radial fibers are immunoreactive for Transitin (arrowheads in C, D).Scale bar, 100 µm.
(3.51 MB TIF)

Figure S7 GFP-labeled cells express a neuronal marker in *Dbx1*-overexpressed pallium. A bipolar-shaped cell is immunoreacitive for TuJ1.
(1.65 MB TIF)
