# Supplementary figures and images for: Patterns of Neurogenesis and Amplitude of Reelin Expression Are Essential for Making a Mammalian-Type Cortex
Source: PLoS One. 2008 Jan 16;3(1):e1454. doi: 10.1371/journal.pone.0001454 (PMC2175532; doi:10.1371/journal.pone.0001454)

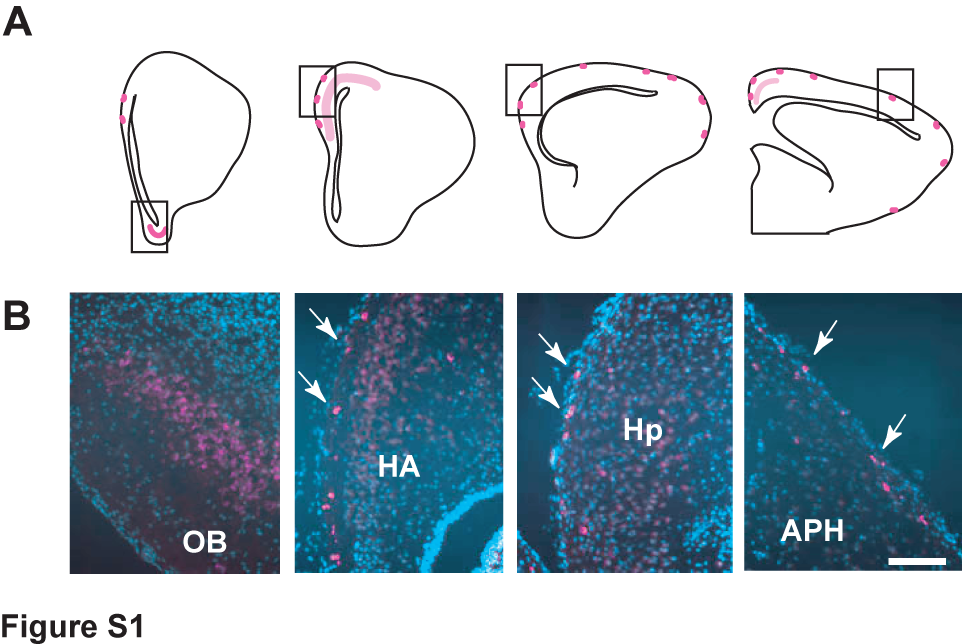

Supplement: Figure S1 — Expression patterns of Reelin in E10 quail pallium. (A) Coronal sections of the quail telencephalon illustrating Reelin expression (magenta). (B) Immunostaining with anti-Reelin antibody in E10 quail telencephalon. Arrows indicate Reelin-positive cells distributed at the pial surface. In later stages, mitral cells in the olfactory bulb (OB), and some neurons in the hyperpallium apicale (HA) and hippocampus (Hp) become to express Reelin, as previously reported in chick embryos [21]. APH: area parahippocampalis. Scale bar: 50 µm. (1.28 MB TIF) [file pone.0001454.s001.tif]

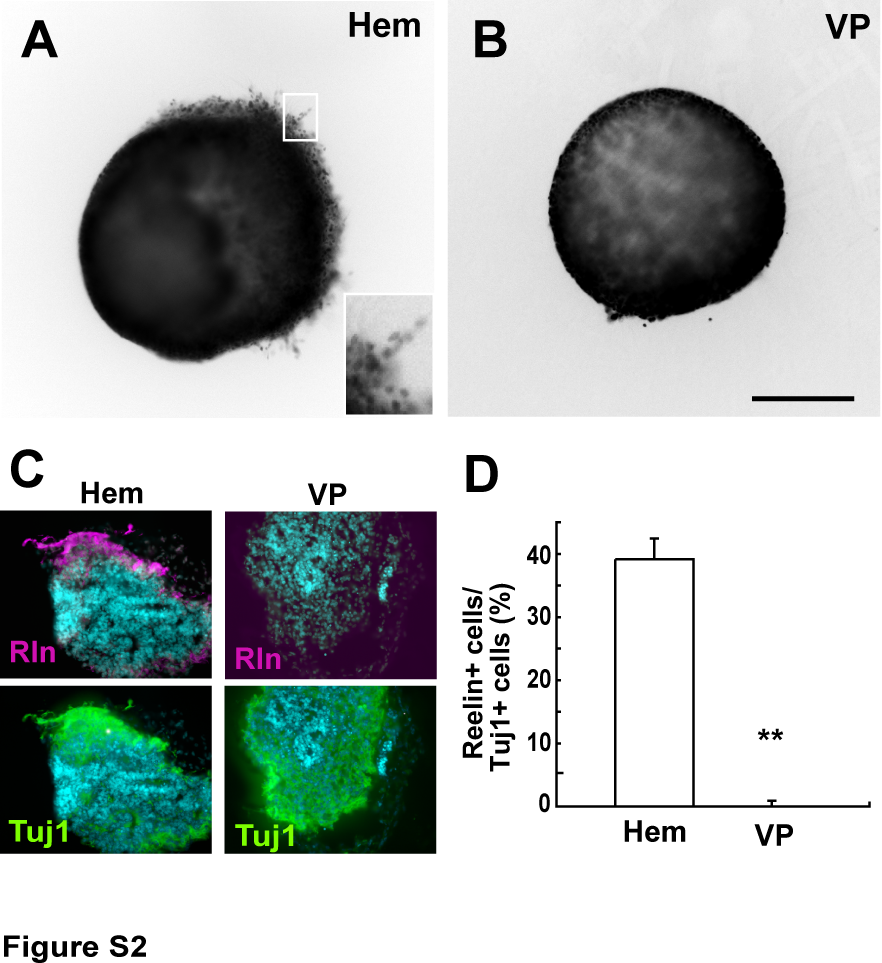

Supplement: Figure S2 — Reelin expression in explant culture. (A, B) Matrigel culture of the E3 chicken cortical hem (A) and ventral pallium (B). After 48 hours of culture, cells migrate out of the hem (inset in A), but not out of the ventral pallium (B). (C) Immunostaining of explants with anti-Reelin and anti-β III tubulin antibodies. (D) The number of Reelin-positive cells in explants. Compared with hem explants, Reelin-positive cells are rarely appeared in the ventral pallium explants. Asterisks indicate statistical significance (p<0.01, t-test). (2.03 MB TIF) [file pone.0001454.s002.tif]

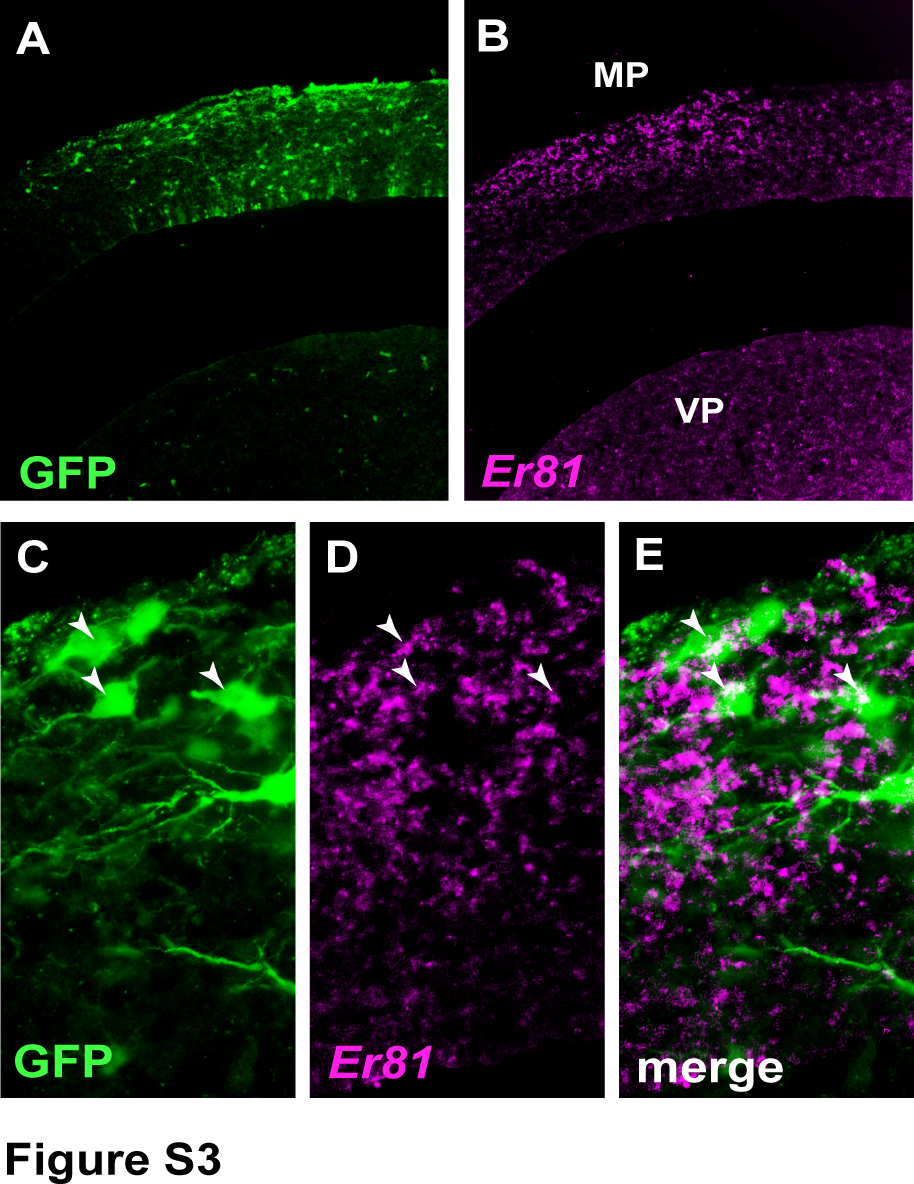

Supplement: Figure S3 — Expression of Er81 in the medial pallium-derived cells. (A–E) Expression of GFP (A, C) and Er81 (B, D) in an E10 embryo in which GFP-plasmid is electroporated into the medial pallium. In situ hybridization with Er81 probe and immunohistochemistry with anti-GFP antibody are performed on same sections. (2.40 MB TIF) [file pone.0001454.s003.tif]

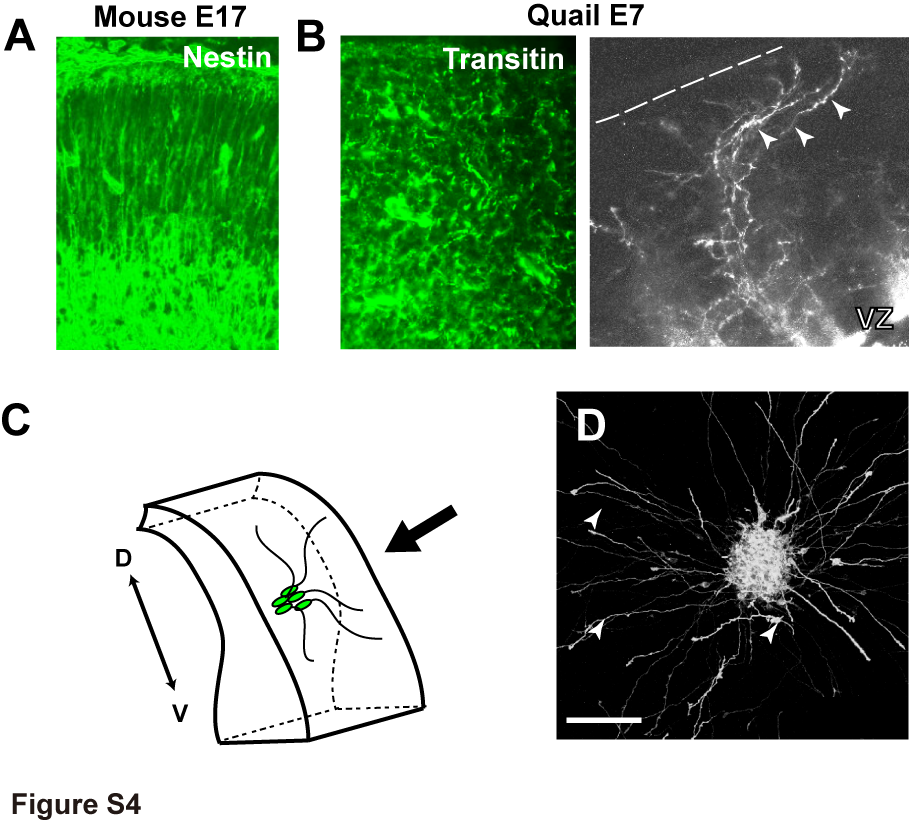

Supplement: Figure S4 — Curved projections of radial glial fibers in the developing quail pallium. (A) Immunostaining with anti-Nestin antibody indicating straight extension of the mouse radial fibers. (B) Immunostaining with anti-Transitin antibody, recognizing an intermediate filament, reveals a mesh-form organization of the quail radial fibers. DiI labeling shows curved extension of each radial fiber in the developing quail pallium. VZ, ventricular zone. (C and D) An image of radial fibers in a flat-mounted quail brain. Confocal microscopic analysis from the pial surface shows multi-directional extension of radial fibers in the developing quail pallium (arrowheads in D). Scale bars, 100 µm. (1.36 MB TIF) [file pone.0001454.s004.tif]

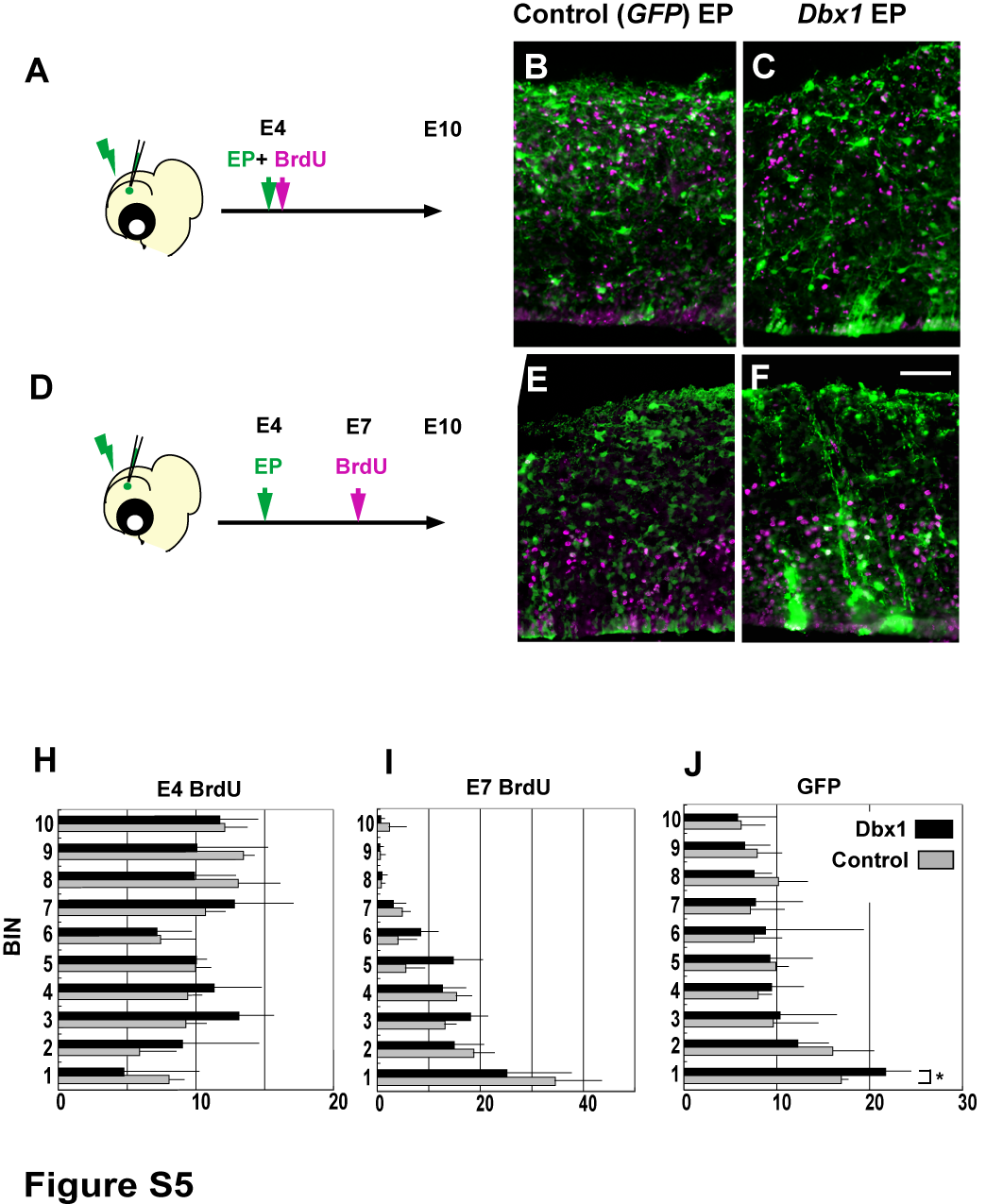

Supplement: Figure S5 — No alterations in birth date-dependent neuronal distribution by Dbx1 overexpression. (A, D) Schematic illustration of schedules of BrdU pulse labeling in electroporated embryos. (B, C, E, F) Distribution of BrdU-positive cells labeled at E4 (B and C) or E7 (E and F) in control (B and C) and Dbx1 overexpressed pallia (E and F). In both cases, the cells incorporated BrdU at E4 are distributed in superficial and deep pallial areas (B and C), whereas the cells labeled at E7 are localized at deep pallial areas (E and F). (H–J) Distribution of BrdU- (H, I) and GFP-positive (J) cells in the control and Dbx1 -overexpressed pallia. No significant changes in the distribution of BrdU-positive cells between the control and Dbx1 -overexpressed pallia (H and I). In contrast, Dbx1 oveexpression increased the number of GFP-positive cells in the ventricular zone (J, BIN1). Asterisk indicates statistical significance (p<0.05, t-test). Scale bar, 100 µm. (1.53 MB TIF) [file pone.0001454.s005.tif]

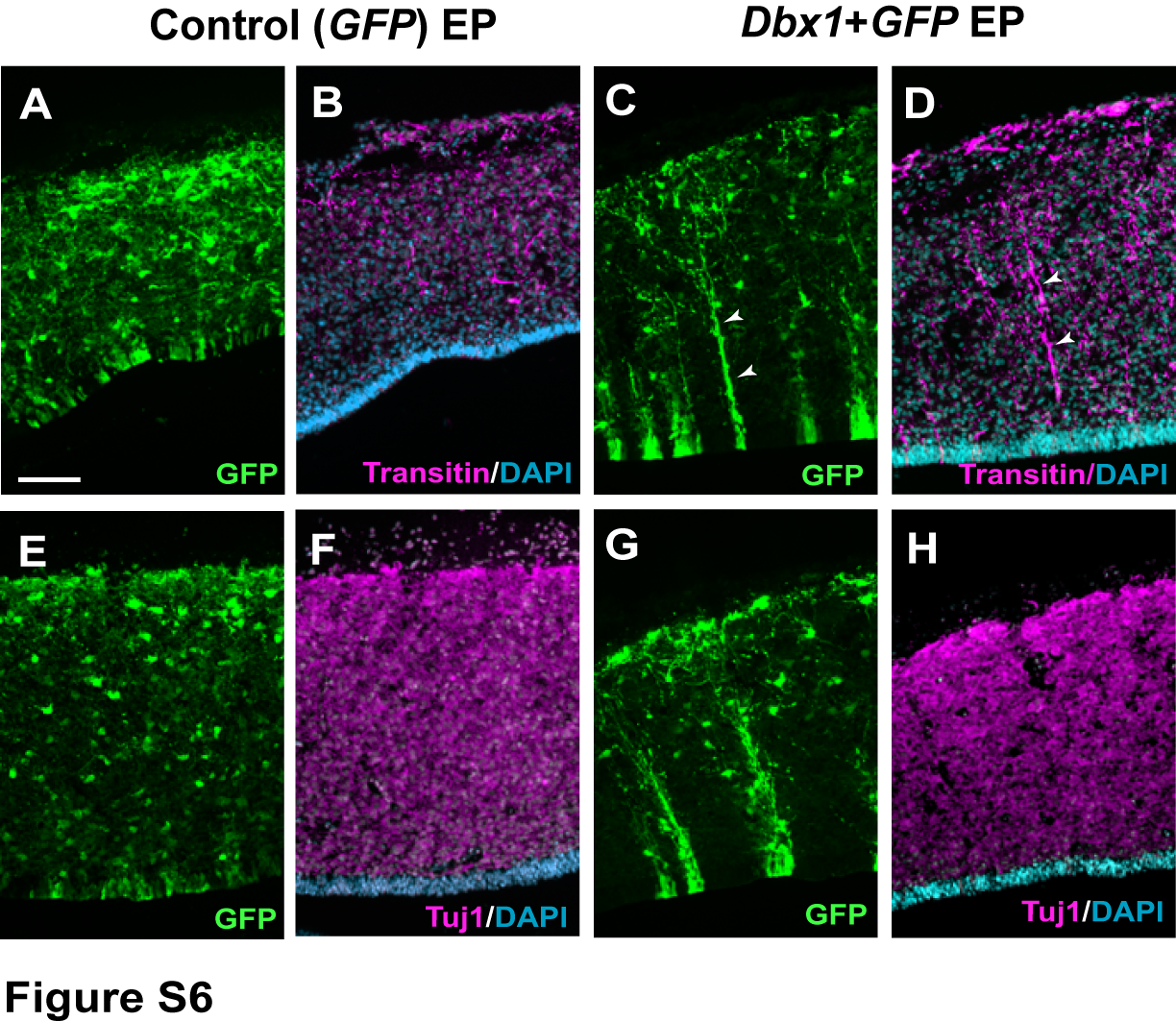

Supplement: Figure S6 — Expression of progenitors and neuronal markers in Dbx1 overexpressed embryos. (A–H) Immunostaining with anti-Transitin (B, D) or anti-β III tubulin (F, H) antibodies of control (A, B, E, F) and Dbx1-overexpressed pallia (C, D, G, H). Elongating radial fibers are immunoreactive for Transitin (arrowheads in C, D).Scale bar, 100 µm. (3.51 MB TIF) [file pone.0001454.s006.tif]

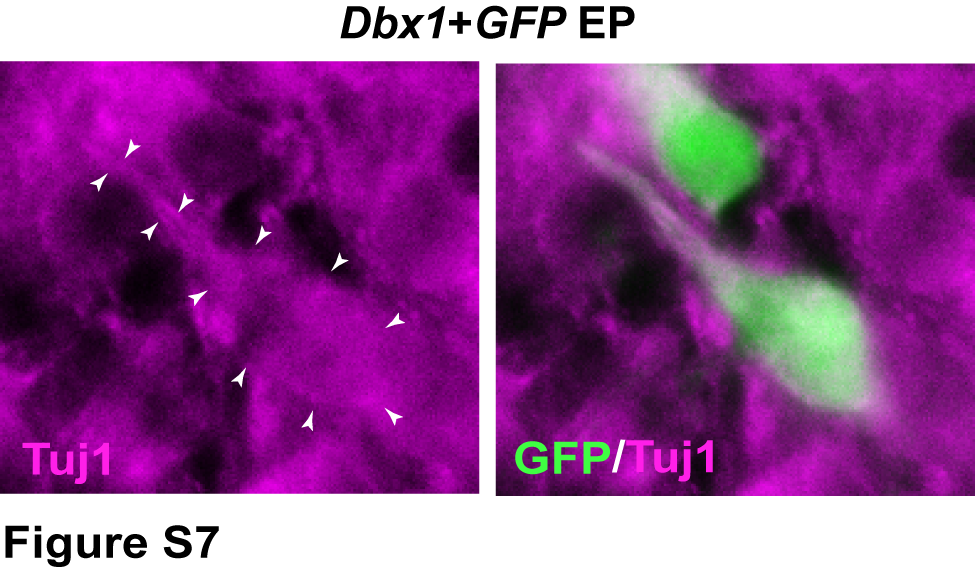

Supplement: Figure S7 — GFP-labeled cells express a neuronal marker in Dbx1-overexpressed pallium. A bipolar-shaped cell is immunoreacitive for TuJ1. (1.65 MB TIF) [file pone.0001454.s007.tif]
